# Supplementary material for: The C-Terminal Repeat Units of SpaA Mediate Adhesion of Erysipelothrix rhusiopathiae to Host Cells and Regulate Its Virulence
Source: Biology (Basel). 2022 Jul 5;11(7):1010. doi: 10.3390/biology11071010 (PMC9311908; doi:10.3390/biology11071010)
Supplement: Supplementary file 1 [file biology-11-01010-s001.zip › biology-1766534-supplementary.pdf]

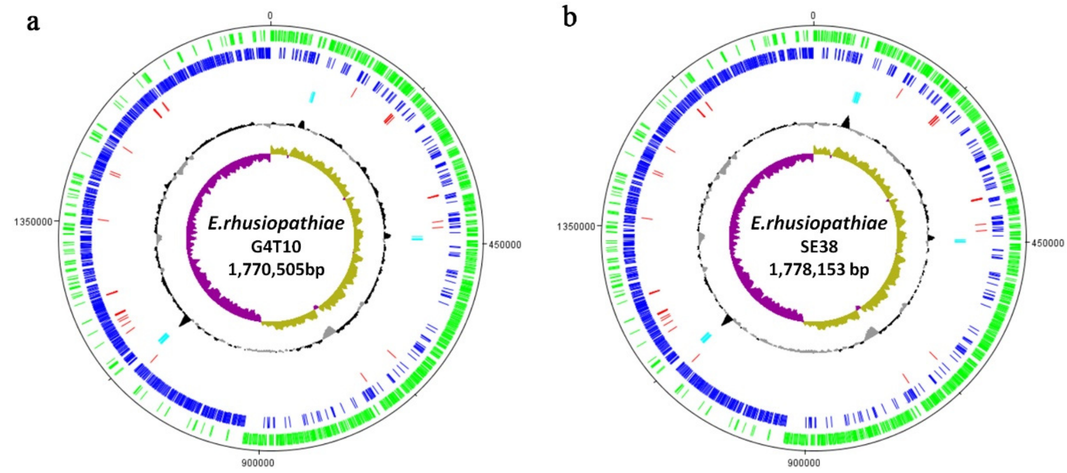

Figure S1: Circular representation of the E. rhusiopathiae G4T10 and E. rhusiopathiae SE38 chromosomes. The coding sequences on the plus and minus strands are shown in circles 1 and circle 2 (outside to inside). tRNA and rRNA are depicted in circles 3 and 4, respectively. GC content (in 10 kb windows) and GC skew curve (10 kb window and 200 bp base steps) are shown in circles 5 and 6, respectively.

```
1    GWIKKDNKWFYIEKSGGMAT    20
2    GWKKVADKWYYLDNTGAIVT    20
3    GWKKVANKWYYLEKSGAMAT    20
4    GWKKVSNKWYYLENSGAMAT    20
5    GWKKVSNKWYYLENSGAMAT    20
6    GWKKVANKWYYLENSGAMAT    20
7    GWKKVSNKWYYLENSGAMAT    20
8    GWKKVANKWYYLDKSGMMVT    20
```

Figure S2: Nucleotide sequence alignment of spaA genes from the six E. rhusiopathiae strains.

Table S1. Summary of bacterial strains and plasmid used in this study

Group	Names	Characteristics and/or function	Source or reference
Bacterial strains	SE38	Virulent strain isolated from a pig with E. rhusiopathiae	This study
	G4T10	Attenuated strain which did not elicit evident virulence in pigs	This study
	Δ*spaA*	The deficient mutant of spa repeat units in SE38	This study
	E. coli DH5	Cloning host for recombinant plasmid	This study
	E. coli BL21(DE3)	Host for over-expressed recombinant spa protein	This study
Plasmid	pSET4s	E. coli- E. rhusiopathiae shuttle vector, themosensitive suicide	This study
	pET28a	Expression vector; KanR	This study

Table S2. Oligonucleotide primers used in this study

Primers	Primers sequence (5–3)[a]	Functions
Δ*spaA*-F	ACCGGATCCTTGTTGAATCCTTAAA	For construction of Δ*spaA* mutant
Δ*spaA*-R	ACCAAGCTTATTCCTATTTATTTTAATGTG	
SpaA-F	AGGGATCCATGAAAAAGAAAAAACACCT	For expression of protein SpaA or SpaA′
SpaA-R	AGCTCGAGCTATTTTAAACTTCCATCGTTCTTA	
P1	TATCAACTTTGCTTTGGGTC	For PCR detection of spaA gene
P2	TCCATCGTTCTTAAATGCA	
P3	CTACGGGAGGCAGCAGTA	For PCR detection of 16S rRNA gene
P4	GTGTACAAGGCCCGAGAA	

[a] Underlined nucleotides denote enzyme restriction sites.

Table S3. General features of *E. rhusiopathiae* strains

Strains	G4T10	SE38	Fujisawa	SY1027	GXBY-1	ATCC19414
Genome Size(bp)	1,770,505	1,778,153	1,787,941	1,752,910	1,876,490	1,746,468#
G+C percentage	36.5%	36.5%	36.6%	36.4%	36.5%	36.0%
CDS number	1,681	1,683	1,678	1,824	1,777	1,631
CDS length(bp)	1,602,906	1,602,249	1,602,939	1,572,462	1,677,114	1,576,866
Average size of CDS (bp)	953	952	955	862	943	966
rRNA operons (16S-23S-5S)	4	5	7	3(+1 5S)	9	2*
tRNA genes	54	55	55	53	57	55

#Total size of four contigs

*Draft genome 3 5S 3 23S 2 16S

Table S4. The list of strain-specific genes

Strains	Locus_tag	COG annotation	Gene product
G4T10	AB984_00403	COG2826L	transposase
G4T10	AB984_00804	-	choline-binding protein
G4T10	AB984_00920	-	DNA-directed DNA polymerase IV
G4T10	AB984_00921	COG0389L	DNA-directed DNA polymerase IV
G4T10	AB984_01316	-	sugar ABC transporter substrate-binding protein
G4T10	AB984_01317	-	sugar ABC transporter substrate-binding protein
G4T10	AB984_01367	-	hypothetical protein
Fujisawa	ERH_00312	COG0513LKJ	DEAD/DEAH box helicase
Fujisawa	ERH_01278	-	hypothetical protein
ATCC19414	HMPREF0357_10164	-	Host cell surface-exposed lipoprotein
ATCC19414	HMPREF0357_10165	-	hypothetical protein
ATCC19414	HMPREF0357_10166	COG3593L	DNA replication and repair protein RecF
ATCC19414	HMPREF0357_10235	COG2801L	transposase
ATCC19414	HMPREF0357_10250	-	hypothetical protein
ATCC19414	HMPREF0357_10889	COG2826L	integrase core domain protein
ATCC19414	HMPREF0357_10901	-	hypothetical protein
ATCC19414	HMPREF0357_10928	COG0019E	Pyridoxal-dependent decarboxylase, pyridoxal binding domain protein
ATCC19414	HMPREF0357_10929	COG1087M	NAD-dependent epimerase/dehydratase family protein
ATCC19414	HMPREF0357_10931	-	glycosyltransferase, group 1 family protein
ATCC19414	HMPREF0357_10932	COG1086MG	polysaccharide biosynthesis protein
ATCC19414	HMPREF0357_10933	-	glycosyltransferase, group 1 family protein
ATCC19414	HMPREF0357_10934	-	hypothetical protein
ATCC19414	HMPREF0357_10958	-	hypothetical protein
ATCC19414	HMPREF0357_10960	-	hypothetical protein
ATCC19414	HMPREF0357_10978	COG2169F	transcriptional regulator, AraC family

ATCC19414	HMPREF0357_10979	-	mucin-desulfating sulfatase
ATCC19414	HMPREF0357_10980	-	carbohydrate ABC transporter, carbohydrate-binding protein
ATCC19414	HMPREF0357_10981	COG1175G	ABC transporter, permease protein
ATCC19414	HMPREF0357_10982	COG0395G	ABC transporter, permease protein
ATCC19414	HMPREF0357_10983	-	1,3-beta-galactosyl-N-acetylhexosamine phosphorylase
ATCC19414	HMPREF0357_10984	-	mucin-desulfating sulfatase
ATCC19414	HMPREF0357_10985	-	N-acetylhexosamine 1-kinase
ATCC19414	HMPREF0357_10986	-	acetyltransferase, GNAT family
ATCC19414	HMPREF0357_11090	-	ArsR family transcriptional regulator
ATCC19414	HMPREF0357_11091	COG2801L	integrase
ATCC19414	HMPREF0357_11092	-	hypothetical protein
ATCC19414	HMPREF0357_11094	-	hypothetical protein
ATCC19414	HMPREF0357_11095	-	hypothetical protein
ATCC19414	HMPREF0357_11096	-	AIPR protein
ATCC19414	HMPREF0357_11097	-	hypothetical protein
ATCC19414	HMPREF0357_11098	-	hypothetical protein
ATCC19414	HMPREF0357_11099	-	hypothetical protein
ATCC19414	HMPREF0357_11100	-	hypothetical protein
ATCC19414	HMPREF0357_11101	-	6-aminohexanoate-dimer hydrolase
ATCC19414	HMPREF0357_11102	-	acetyltransferase, GNAT family
ATCC19414	HMPREF0357_11103	COG3410S	GIY-YIG catalytic domain protein
ATCC19414	HMPREF0357_11104	-	nucleotide pyrophosphohydrolase
ATCC19414	HMPREF0357_11105	COG1715V	restriction endonuclease
ATCC19414	HMPREF0357_11175	COG3886L	helicase
ATCC19414	HMPREF0357_11183	COG1974KT	peptidase S24-like protein
ATCC19414	HMPREF0357_11463	-	LPXTG-motif cell wall anchor domain protein
ATCC19414	HMPREF0357_11464	COG2801L	integrase core domain protein

ATCC19414	HMPREF0357_11465	-	Transposase
ATCC19414	HMPREF0357_11476	-	ArsR family transcriptional regulator
ATCC19414	HMPREF0357_11477	-	hypothetical protein
ATCC19414	HMPREF0357_11591	-	MucBP domain protein
GXBY-1	A2I91_RS00085	-	hypothetical protein
GXBY-1	A2I91_RS02495	-	hypothetical protein
GXBY-1	A2I91_RS02500	-	replication initiator protein A
GXBY-1	A2I91_RS02505	COG1484L	AAA family ATPase
GXBY-1	A2I91_RS02510	-	PcfB family protein
GXBY-1	A2I91_RS02515	COG3645S	antirepressor
GXBY-1	A2I91_RS02525	-	hypothetical protein
GXBY-1	A2I91_RS02530	-	hypothetical protein
GXBY-1	A2I91_RS02535	COG2207K	AraC family transcriptional regulator
GXBY-1	A2I91_RS02540	-	hypothetical protein
GXBY-1	A2I91_RS02545	-	conjugal transfer protein
GXBY-1	A2I91_RS02550	-	hypothetical protein
GXBY-1	A2I91_RS02555	-	hypothetical protein
GXBY-1	A2I91_RS02560	-	PrgI family protein
GXBY-1	A2I91_RS02570	COG0791M	hypothetical protein
GXBY-1	A2I91_RS02575	-	hypothetical protein
GXBY-1	A2I91_RS02580	-	copper amine oxidase
GXBY-1	A2I91_RS02585	-	DUF4366 domain-containing protein
GXBY-1	A2I91_RS02590	COG0550L	DNA topoisomerase III
GXBY-1	A2I91_RS02595	COG0270L	DNA (cytosine-5-)-methyltransferase
GXBY-1	A2I91_RS02600	COG4646KL	DNA helicase
GXBY-1	A2I91_RS02605	-	hypothetical protein
GXBY-1	A2I91_RS02610	COG3655K	transcriptional regulator

GXBY-1	A2I91_RS02675	COG2227H	class I SAM-dependent methyltransferase
GXBY-1	A2I91_RS02620	-	aminoglycoside nucleotidyltransferase ANT(6)-Ia
GXBY-1	A2I91_RS02625	COG0503F	adenine phosphoribosyltransferase
GXBY-1	A2I91_RS02630	COG1708R	ANT(9) family aminoglycoside nucleotidyltransferase Spw
GXBY-1	A2I91_RS02640	-	hypothetical protein
GXBY-1	A2I91_RS02645	-	hypothetical protein
GXBY-1	A2I91_RS02650	COG0488R	ABC-F type ribosomal protection protein Lsa(E)]
GXBY-1	A2I91_RS02655	-	lincosamide nucleotidyltransferase Lnu(B)]
GXBY-1	A2I91_RS02660	-	recombinase
GXBY-1	A2I91_RS02665	-	XRE family transcriptional regulator
GXBY-1	A2I91_RS02670	-	hypothetical protein
GXBY-1	A2I91_RS02680	-	aminoglycoside nucleotidyltransferase ANT(6)-Ia
GXBY-1	A2I91_RS02685	COG0454KR	streptothricin N-acetyltransferase Sat4
GXBY-1	A2I91_RS02690	COG3231J	aminoglycoside O-phosphotransferase APH(3')-IIIa
GXBY-1	A2I91_RS02695	COG2378K	HTH domain-containing protein
GXBY-1	A2I91_RS02705	COG1985H	RibD family protein
GXBY-1	A2I91_RS02710	-	hypothetical protein
GXBY-1	A2I91_RS02715	-	hypothetical protein
GXBY-1	A2I91_RS02720	COG4974L	transposase
GXBY-1	A2I91_RS02725	-	excisionase
GXBY-1	A2I91_RS02730	-	helix-turn-helix domain-containing protein
GXBY-1	A2I91_RS02735	-	sigma-70 family RNA polymerase sigma factor
GXBY-1	A2I91_RS02740	-	XRE family transcriptional regulator
GXBY-1	A2I91_RS02745	COG0480J	tetracycline resistance ribosomal protection protein Tet(M)]
GXBY-1	A2I91_RS02750	-	conjugal transfer protein
GXBY-1	A2I91_RS02755	COG0741M	peptidase P60
GXBY-1	A2I91_RS02760	-	membrane protein

GXBY-1	A2I91_RS02765	-	ATP/GTP-binding protein
GXBY-1	A2I91_RS02770	-	conjugal transfer protein
GXBY-1	A2I91_RS02775	COG4734R	antirestriction protein ArdA
GXBY-1	A2I91_RS02780	-	hypothetical protein
GXBY-1	A2I91_RS02785	COG2946L	XRE family transcriptional regulator
GXBY-1	A2I91_RS02790	COG1674D	DNA translocase FtsK
GXBY-1	A2I91_RS02795	-	DUF961 domain-containing protein
GXBY-1	A2I91_RS02800	-	DUF961 domain-containing protein
GXBY-1	A2I91_RS02805	-	hypothetical protein
GXBY-1	A2I91_RS02810	-	hypothetical protein
GXBY-1	A2I91_RS02815	-	DUF4868 domain-containing protein
GXBY-1	A2I91_RS02820	COG3843U	endonuclease
GXBY-1	A2I91_RS02825	-	plasmid mobilization relaxosome protein MobC
GXBY-1	A2I91_RS02830	-	ATP-binding protein
GXBY-1	A2I91_RS02835	-	hypothetical protein
GXBY-1	A2I91_RS02840	COG2856E	ImmA/IrrE family metallo-endopeptidase
GXBY-1	A2I91_RS02845	-	XRE family transcriptional regulator
GXBY-1	A2I91_RS02850	-	hypothetical protein
GXBY-1	A2I91_RS02855	-	sigma-70 family RNA polymerase sigma factor
GXBY-1	A2I91_RS02860	-	hypothetical protein
GXBY-1	A2I91_RS02865	COG1961L	recombinase
GXBY-1	A2I91_RS02870	COG1961L	recombinase
GXBY-1	A2I91_RS02875	COG1961L	recombinase
GXBY-1	A2I91_RS02880	-	restriction endonuclease
GXBY-1	A2I91_RS09020	COG3655K	transcriptional regulator
SY1027	K210_RS00005	COG1118P	sugar ABC transporter ATP-binding protein
SY1027	K210_RS00080	-	hypothetical protein

SY1027	K210_RS00110	COG0406G	phosphoglycerate mutase family protein
SY1027	K210_RS00120	-	lipoyltransferase and lipoate-protein ligase
SY1027	K210_RS00270	-	iron-sulfur-binding protein
SY1027	K210_RS00285	COG0013J	alanyl-tRNA synthetase
SY1027	K210_RS00345	COG4720S	membrane protein
SY1027	K210_RS00350	COG0481M	elongation factor 4
SY1027	K210_RS00460	COG0522J	30S ribosomal protein S4
SY1027	K210_RS00470	COG1104E	cysteine desulfurase
SY1027	K210_RS00510	COG0550L	DNA topoisomerase I
SY1027	K210_RS00545	-	UDP-N-acetylenolpyruvoylglucosamine reductase
SY1027	K210_RS00695	-	glutamyl aminopeptidase
SY1027	K210_RS00780	COG0443O	molecular chaperone DnaK
SY1027	K210_RS00965	-	alanine racemase
SY1027	K210_RS01135	COG4586R	multidrug ABC transporter
SY1027	K210_RS01150	-	thymidylate synthase
SY1027	K210_RS01170	-	transposase
SY1027	K210_RS01175	-	hypothetical protein
SY1027	K210_RS01180	-	phage infection protein
SY1027	K210_RS01190	-	hypothetical protein
SY1027	K210_RS01195	-	bacteriocin transporter
SY1027	K210_RS01305	COG0269G	3-keto-L-gulonate-6-phosphate decarboxylase
SY1027	K210_RS01325	-	NAD-dependent malic enzyme 4
SY1027	K210_RS01685	-	hypothetical protein
SY1027	K210_RS01705	-	accessory gene regulator C
SY1027	K210_RS01820	-	histidine kinase
SY1027	K210_RS01870	-	hypothetical protein
SY1027	K210_RS01885	COG1058R	damage-inducible protein CinA

SY1027	K210_RS01925	-	transcriptional regulator
SY1027	K210_RS01945	-	hypothetical protein
SY1027	K210_RS01980	COG3475M	lipopolysaccharide cholinephosphotransferase
SY1027	K210_RS02090	COG1154HI	1-deoxy-D-xylulose-5-phosphate synthase
SY1027	K210_RS02115	-	hypothetical protein
SY1027	K210_RS02135	COG0130J	tRNA pseudouridine synthase B
SY1027	K210_RS02220	-	Holliday junction DNA helicase RuvB
SY1027	K210_RS02255	-	transposase
SY1027	K210_RS07350	COG2826L	transposase
SY1027	K210_RS02330	-	GTP-binding protein
SY1027	K210_RS02345	COG0187L	DNA topoisomerase IV, B subunit
SY1027	K210_RS02435	COG3601S	Riboflavin transporter RibU
SY1027	K210_RS02495	-	SAM-dependent methyltransferase
SY1027	K210_RS02505	-	DNA primase
SY1027	K210_RS02560	-	PBP superfamily domain protein
SY1027	K210_RS02620	COG1187J	pseudouridylate synthase
SY1027	K210_RS02635	COG0213F	pyrimidine-nucleoside phosphorylase
SY1027	K210_RS02660	COG0612R	peptidase M16
SY1027	K210_RS02730	COG0237H	dephospho-CoA kinase
SY1027	K210_RS02735	COG0266L	DNA-formamidopyrimidine glycosylase
SY1027	K210_RS02760	-	cell shape-determining protein
SY1027	K210_RS02790	COG0036G	ribulose-phosphate 3-epimerase
SY1027	K210_RS02810	-	rRNA methyltransferase
SY1027	K210_RS02825	-	fibronectin-binding protein
SY1027	K210_RS02840	-	hypothetical protein
SY1027	K210_RS02850	-	Type IV leader peptidase family protein
SY1027	K210_RS02895	COG1269C	V-type ATPase subunit I

SY1027	K210_RS02900	-	putative ATP synthase, subunit C
SY1027	K210_RS02915	COG0205G	phosphofructokinase
SY1027	K210_RS02920	-	ABC transporter ATP-binding protein
SY1027	K210_RS03010	COG0566J	RNA methyltransferase
SY1027	K210_RS03015	COG0060J	isoleucyl-tRNA synthetase
SY1027	K210_RS03050	COG0474P	magnesium-transporting ATPase
SY1027	K210_RS03065	-	exonuclease SbcC
SY1027	K210_RS03085	COG0153G	galactokinase
SY1027	K210_RS03095	COG1129G	D-ribose transporter ATP-binding protein
SY1027	K210_RS03140	COG0544O	trigger factor
SY1027	K210_RS03145	-	hypothetical protein
SY1027	K210_RS03180	COG1193L	DNA strand exchange inhibitor protein
SY1027	K210_RS03215	-	multidrug transporter
SY1027	K210_RS03245	COG4152R	ABC transporter ATP-binding protein
SY1027	K210_RS03260	-	YhaG family tryptophan uptake permease
SY1027	K210_RS03340	-	acetyltransferase
SY1027	K210_RS03400	-	transporter
SY1027	K210_RS03410	COG1122P	cobalt ABC transporter ATP-binding protein
SY1027	K210_RS03500	COG0201U	preprotein translocase subunit SecY
SY1027	K210_RS03525	COG0096J	30S ribosomal protein S8
SY1027	K210_RS03535	COG0094J	50S ribosomal protein L5
SY1027	K210_RS03545	COG0093J	50S ribosomal protein L14
SY1027	K210_RS03690	-	ABC transporter permease
SY1027	K210_RS03815	-	hyaluronidase
SY1027	K210_RS03855	-	oligohyaluronate lyase
SY1027	K210_RS04010	COG1011R	haloacid dehalogenase
SY1027	K210_RS04020	-	esterase

SY1027	K210_RS04040	-	multidrug transporter MATE
SY1027	K210_RS04050	-	cell wall anchor
SY1027	K210_RS08760	-	restriction endonuclease
SY1027	K210_RS04220	-	recombination protein F
SY1027	K210_RS04225	-	collagen adhesion protein
SY1027	K210_RS04230	-	hypothetical protein
SY1027	K210_RS04235	COG0338L	Site-specific DNA methylase
SY1027	K210_RS04240	-	SpoVT/AbrB domain-containing protein
SY1027	K210_RS04245	-	hypothetical protein
SY1027	K210_RS04250	-	hypothetical protein
SY1027	K210_RS04255	-	hypothetical protein
SY1027	K210_RS04260	-	hypothetical protein
SY1027	K210_RS04265	-	hypothetical protein
SY1027	K210_RS04270	-	hypothetical protein
SY1027	K210_RS08650	COG0739M	peptidase M23
SY1027	K210_RS04285	-	hypothetical protein
SY1027	K210_RS08765	-	hypothetical protein
SY1027	K210_RS04295	-	hypothetical protein
SY1027	K210_RS04300	-	hypothetical protein
SY1027	K210_RS08660	COG0863L	Modification methylase HindIII
SY1027	K210_RS04310	COG0863L	BslIM
SY1027	K210_RS04315	COG0863L	BslIM
SY1027	K210_RS04320	-	hypothetical protein
SY1027	K210_RS04325	-	hypothetical protein
SY1027	K210_RS04330	-	DNA primase
SY1027	K210_RS04335	-	hypothetical protein
SY1027	K210_RS04345	COG1191K	RNA polymerase sigma factor SigD

SY1027	K210_RS04350	-	anaerobic benzoate catabolism transcriptional regulator
SY1027	K210_RS04355	-	ICEBs1 excisionase
SY1027	K210_RS04360	COG0582L	phage integrase family protein
SY1027	K210_RS04365	-	hypothetical protein
SY1027	K210_RS04370	-	hypothetical protein
SY1027	K210_RS04420	-	hypothetical protein
SY1027	K210_RS04480	-	ATPase
SY1027	K210_RS08665	-	acetyltransferase, GNAT family
SY1027	K210_RS04535	COG0789K	MerR family transcriptional regulator
SY1027	K210_RS04620	COG1454C	bifunctional acetaldehyde-CoA/alcohol dehydrogenase
SY1027	K210_RS04680	COG2365T	aldo/keto reductase
SY1027	K210_RS04755	-	hypothetical protein
SY1027	K210_RS04765	-	ABC transporter
SY1027	K210_RS04805	-	bacteriocin transporter
SY1027	K210_RS04830	-	hypothetical protein
SY1027	K210_RS04875	-	sugar phosphate isomerase
SY1027	K210_RS04880	COG3933K	PRD domain protein
SY1027	K210_RS04940	COG1071C	pyruvate dehydrogenase E1 subunit alpha
SY1027	K210_RS05050	-	hypothetical protein
SY1027	K210_RS05055	-	hypothetical protein
SY1027	K210_RS05075	-	collagen-binding protein
SY1027	K210_RS06875	-	transposase
SY1027	K210_RS05130	COG0399M	aminotransferase DegT
SY1027	K210_RS05135	-	polysaccharide biosynthesis protein
SY1027	K210_RS05165	-	acetyl transferase
SY1027	K210_RS05170	COG3274S	acetyl transferase
SY1027	K210_RS05175	-	peptidase

SY1027	K210_RS05255	-	internalin
SY1027	K210_RS05290	COG3845R	heme ABC transporter ATP-binding protein
SY1027	K210_RS05325	-	choline/ethanolamine kinase
SY1027	K210_RS05345	-	ABC transporter permease
SY1027	K210_RS05485	COG0018J	arginyl-tRNA synthetase
SY1027	K210_RS05525	-	FMN reductase
SY1027	K210_RS05550	COG0692L	uracil-DNA glycosylase
SY1027	K210_RS05635	COG1481S	Putative sporulation transcription regulator WhiA
SY1027	K210_RS05695	COG2826L	transposase
SY1027	K210_RS05725	COG1186J	peptide chain release factor 2
SY1027	K210_RS05815	-	epimerase
SY1027	K210_RS05975	COG0025P	transporter, CPA2 family
SY1027	K210_RS06040	-	PEP phosphonomutase
SY1027	K210_RS06060	-	hypothetical protein
SY1027	K210_RS06160	-	thermostable hemolysin delta-VPH
SY1027	K210_RS06245	COG0030J	dimethyladenosine transferase
SY1027	K210_RS06335	-	LPXTG-motif cell wall anchor domain protein
SY1027	K210_RS06380	-	cell surface protein
SY1027	K210_RS06480	COG1105G	fructose-1-phosphate kinase
SY1027	K210_RS06680	COG0803P	manganese transporter
SY1027	K210_RS06690	COG1108P	manganese ABC transporter permease
SY1027	K210_RS06740	-	sulfatase
SY1027	K210_RS06760	-	hypothetical protein
SY1027	K210_RS06785	-	GNAT family acetyltransferase
SY1027	K210_RS08680	-	A/G-specific adenine glycosylase
SY1027	K210_RS06890	-	hypothetical protein
SY1027	K210_RS06955	-	NAD(+) synthase

SY1027	K210_RS06970	-	ABC transporter
SY1027	K210_RS08805	-	transposase
SY1027	K210_RS07875	COG2801L	integrase core domain protein
SY1027	K210_RS08810	COG0557K	ribonuclease R
SY1027	K210_RS07095	-	GCN5 family acetyltransferase
SY1027	K210_RS07100	COG4684S	membrane protein
SY1027	K210_RS07185	COG1916S	pheromone shutdown protein
SY1027	K210_RS07215	COG0086K	DNA-directed RNA polymerase subunit beta'
SY1027	K210_RS08690	COG2207K	AraC family transcriptional regulator
SY1027	K210_RS07340	COG0584C	glycerophosphodiester phosphodiesterase
SY1027	K210_RS07365	-	transcriptional regulator, TetR family
SY1027	K210_RS07420	-	hypothetical protein
SY1027	K210_RS07430	-	type III pantothenate kinase
SY1027	K210_RS07445	-	hypothetical protein
SY1027	K210_RS07620	COG1283P	Na/Pi cotransporter
SY1027	K210_RS07650	COG1263G	PTS glucose transporter subunit IIABC
SY1027	K210_RS08015	COG2391R	membrane protein
SY1027	K210_RS08700	-	histidine kinase
SY1027	K210_RS08080	COG1114E	branched-chain amino acid ABC transporter substrate-binding protein
SY1027	K210_RS08085	-	hypothetical protein
SY1027	K210_RS08120	-	hypothetical protein
SY1027	K210_RS08250	-	CoA-binding protein
SY1027	K210_RS08260	-	transcriptional regulator
SY1027	K210_RS08290	COG0209F	ribonucleotide reductase
SY1027	K210_RS08335	COG2183K	hypothetical protein
SY1027	K210_RS08415	-	benzene 1,2-dioxygenase
SY1027	K210_RS08565	COG1164E	peptidase M3

SY1027	K210_RS08575	-	ABC transporter substrate-binding protein
SY1027	K210_RS08585	COG1101R	ABC transporter

Table S5 Virulence factors in six *Erysipelothrix rhusiopathiae* strains

VFDB_ID	Annotation	Locus tags					
		G4T10	SE38	Fujisawa	GXBY-1	SY1027	ATCC19414
**Adhesion**							
**CD0194(AI317)**	60 kDa chaperonin	AB984_01338	AB985_01311	ERH_RS06540	A2I91_RS02965	K210_RS04095	HMPREF0357_11107
**SSU98_1513(AI215)**	phosphopyruvate hydratase	AB984_01402	AB985_01374	ERH_RS06845	A2I91_RS02270	K210_RS04560	HMPREF0357_11052
**MPN665(AI171)**	elongation factor Tu	AB984_01239	AB985_01212	ERH_RS06050	A2I91_RS03465	K210_RS03610	HMPREF0357_11199
**BAC67690(AI162)**	rspB, rhusiopathiae surface protein B	AB984_00702	AB985_00689	ERH_RS03415	A2I91_RS06165	K210_RS01025	HMPREF0357_10208
**M28_Spy0227(AI210)**	glyceraldehyde-3-phosphate dehydrogenase	AB984_01614	AB985_01584	ERH_RS07885	A2I91_RS01230	K210_RS05615	HMPREF0357_10840
**lmo1634(AI351)**	lap, bifunctional acetaldehyde-CoA/alcohol dehydrogenase	AB984_01415	AB985_01386	ERH_RS06905	A2I91_RS02210	K210_RS04620	HMPREF0357_11042
**BAC67689(AI161)**	rspA, rhusiopathiae surface protein A	AB984_00701, AB984_00700	AB985_00688	ERH_RS03410	A2I91_RS06170	K210_RS01020	HMPREF0357_10207
**SAG1594(AI186)**	scpB, segregation and condensation protein B	AB984_01034	AB985_01012	ERH_RS05020	A2I91_RS04555	K210_RS02625	HMPREF0357_10531
**lmo1829(AI163)**	hypothetical protein	AB984_01076	AB985_01053	ERH_RS05220	A2I91_RS04355	K210_RS02825	HMPREF0357_10571
**CT396(AI392)**	molecular chaperone DnaK	AB984_00604	AB985_00593	ERH_RS02935	A2I91_RS06645	K210_RS00780	HMPREF0357_10155
**LA_4119(AI240)**	ligA, NAD dependent DNA ligase	AB984_00356	AB985_00351	ERH_RS01725	A2I91_RS07855	K210_RS08180	HMPREF0357_11388
**-**	internalin-like protein	AB984_01544	AB985_01514	ERH_RS07530	A2I91_RS01585	K210_RS05255	HMPREF0357_10902
**-**	Surface protective antigen A	AB984_00104	AB985_00101	ERH_RS00500	A2I91_RS09085	K210_RS06960	HMPREF0357_11626
**Secretion**							
**lpg1962(SS047)**	lirB, peptidyl-prolyl cis-trans isomerase (rotamase)	AB984_00462	AB985_00455	ERH_RS02245	A2I91_RS07335	K210_RS00090	HMPREF0357_10017
**PA0073(SS178)**	tagT, ATP-binding component of ABC transporter	AB984_01531	AB985_01501	ERH_RS07465	A2I91_RS01650	K210_RS05190	HMPREF0357_10917
**CV_2635(SS016)**	armR, two-component response regulator	AB984_01562, AB984_01023, AB984_00332	AB985_01533, AB985_01001, AB985_00328	ERH_RS07630, ERH_RS04965, ERH_RS01605	A2I91_RS01485, A2I91_RS04610, A2I91_RS07975	K210_RS05360, K210_RS02570, K210_RS08060	HMPREF0357_10880 , HMPREF0357_10520

							,HMPREF0357_1141 2
**-**	leucine-rich repeat protein	AB984_00762	AB985_00748	ERH_RS03705	A2I91_RS05860	K210_RS01320	HMPREF0357_10267
**-**	Carbohydrate ABC transporter, substrate-binding protein	AB984_01317, AB984_01316	AB985_01290	ERH_RS06435	A2I91_RS03070	K210_RS03990	HMPREF0357_11128
**Iron**							
**PSPTO_2604(IA005)**	ybtP, ABC transporter ATP-binding/permease	AB984_01560	AB985_01531	ERH_RS07620	A2I91_RS01495	K210_RS05350	HMPREF0357_10882
**AAR12524(IA024)**	fatD, ferric anguibactin transport protein	AB984_01437	AB985_01407	ERH_RS07010	A2I91_RS02105	K210_RS04725	HMPREF0357_11021
**PA4706(IA049)**	phuV, hemin importer ATP-binding subunit	AB984_00843	AB985_00826	ERH_RS04100	A2I91_RS05475	K210_RS01715	HMPREF0357_10347
**PSEEN2494(IA004)**	bauE, ferric siderophore ABC transporter ATP-binding protein BauE	AB984_00935	AB985_00915	ERH_RS04535	A2I91_RS05040	K210_RS02145	HMPREF0357_10437
**AAR12526(IA024)**	fatB, ferric anguibactin transport protein	AB984_01440	AB985_01410	ERH_RS07025	A2I91_RS02090	K210_RS04740	HMPREF0357_11018
